# 

**Published:** 1910-09

**Authors:** 


					﻿Gastroenterostomy For Drunkenness (Dr. Kenney’s Operation).
Kenney's Famous Sanatorium: The “Indiana Plan.”
■L	&
i	X	BI-
SEPTEMBER, 1910.
No. 3.
law
-I- E X A S
MEDICAL
JOURNAL
"Red Back”
PUBLISHED AT AUSTIN, TEXAS, BY
F. E. DANIEL, M. D., - - - - PjppriW
PRINTED BY THE VON BOECKMANN-JONA C
Subscription, 81.00 a Year, in Advance. -	a^ge (&pfc£, TofWits
Entered at the Postoffice at Austin, Texas, as second class mail matter.
				

